# Exploring machine learning strategies for predicting cardiovascular disease risk factors from multi-omic data

**DOI:** 10.1186/s12911-024-02521-3

**Published:** 2024-05-02

**Authors:** Gabin Drouard, Juha Mykkänen, Jarkko Heiskanen, Joona Pohjonen, Saku Ruohonen, Katja Pahkala, Terho Lehtimäki, Xiaoling Wang, Miina Ollikainen, Samuli Ripatti, Matti Pirinen, Olli Raitakari, Jaakko Kaprio

**Affiliations:** 1 Institute for Molecular Medicine Finland (FIMM), HiLIFE, University of Helsinki, Helsinki, Finland; 2 Centre for Population Health Research, University of Turku and Turku University Hospital, Turku, Finland; 3 Research Centre of Applied and Preventive Cardiovascular Medicine, University of Turku, Turku, Finland; 4 Research Program in Systems Oncology, University of Helsinki, Helsinki, Finland; 5 Paavo Nurmi Centre & Unit for Health and Physical Activity, University of Turku, Turku, Finland; 6 Department of Clinical Chemistry, Fimlab Laboratories, and Finnish Cardiovascular Research Center - Tampere, Faculty of Medicine and Health Technology, Tampere University, 33520 Tampere, Finland; 7 Georgia Prevention Institute, Medical College of Georgia, Augusta University, Augusta, GA USA; 8 Minerva Foundation Institute for Medical Research, Helsinki, Finland; 9 Public Health, Faculty of Medicine, University of Helsinki, Helsinki, Finland; 10 Broad Institute of MIT and Harvard, Cambridge, MA USA; 11 Department of Mathematics and Statistics, University of Helsinki, Helsinki, Finland; 12 Department of Clinical Physiology and Nuclear Medicine, Turku University Hospital, Turku, Finland

**Keywords:** Multi-omics, Autoencoders, Meta-learners, Cardiovascular disease, Blood pressure, Hypertension, Diastolic function, Imbalanced design, Predictions

## Abstract

**Background:**

Machine learning (ML) classifiers are increasingly used for predicting cardiovascular disease (CVD) and related risk factors using omics data, although these outcomes often exhibit categorical nature and class imbalances. However, little is known about which ML classifier, omics data, or upstream dimension reduction strategy has the strongest influence on prediction quality in such settings. Our study aimed to illustrate and compare different machine learning strategies to predict CVD risk factors under different scenarios.

**Methods:**

We compared the use of six ML classifiers in predicting CVD risk factors using blood-derived metabolomics, epigenetics and transcriptomics data. Upstream omic dimension reduction was performed using either unsupervised or semi-supervised autoencoders, whose downstream ML classifier performance we compared. CVD risk factors included systolic and diastolic blood pressure measurements and ultrasound-based biomarkers of left ventricular diastolic dysfunction (LVDD; E/e' ratio, E/A ratio, LAVI) collected from 1,249 Finnish participants, of which 80% were used for model fitting. We predicted individuals with low, high or average levels of CVD risk factors, the latter class being the most common. We constructed multi-omic predictions using a meta-learner that weighted single-omic predictions. Model performance comparisons were based on the F1 score. Finally, we investigated whether learned omic representations from pre-trained semi-supervised autoencoders could improve outcome prediction in an external cohort using transfer learning.

**Results:**

Depending on the ML classifier or omic used, the quality of single-omic predictions varied. Multi-omics predictions outperformed single-omics predictions in most cases, particularly in the prediction of individuals with high or low CVD risk factor levels. Semi-supervised autoencoders improved downstream predictions compared to the use of unsupervised autoencoders. In addition, median gains in Area Under the Curve by transfer learning compared to modelling from scratch ranged from 0.09 to 0.14 and 0.07 to 0.11 units for transcriptomic and metabolomic data, respectively.

**Conclusions:**

By illustrating the use of different machine learning strategies in different scenarios, our study provides a platform for researchers to evaluate how the choice of omics, ML classifiers, and dimension reduction can influence the quality of CVD risk factor predictions.

**Supplementary Information:**

The online version contains supplementary material available at 10.1186/s12911-024-02521-3.

## Background

Cardiovascular disease (CVD) is one of the leading causes of death in the world and its prevalence has been increasing globally over the past three decades [1]. Substantial genetic components associated with CVDs have been identified [2, 3], but the linking of knowledge gained at different molecular levels remains incomplete. The use of integrative modelling in genomic studies, commonly referred to as multi-omics modelling, has broad but still largely unrealised potential for both the diagnosis and discovery of the aetiology of CVD [4, 5]. Interest in studying risk factors for CVDs has also increased, as generating multi-omic data in observational cohorts with a sufficient number of CVD cases can be challenging. In addition, a large number of CVDs share common risk factors that can be easily measured non-invasively, such as blood pressure. A deeper understanding of CVD risk factors and, for example, which omic layers best predict individuals at increased risk, would enable the better identification of individuals at risk for future cardiovascular outcomes.

The integration of omics data into multimodal modelling is a rapidly expanding area of research whose (dis)advantages over single-omics approaches have already been discussed [6], despite its relatively modest utilization in cardiovascular research [7, 8]. The complexity of multi-omics approaches may cause challenges at methodological level (e.g. omics pre-processing, small sample sizes, imbalanced study designs, high dimensions) and restrict the replicability of multi-omics models. The use of integrative strategies may also raise additional challenges in predictive settings, such as balancing model interpretability and model performance: the search for a high predictive performance often leads to the use of advanced methods (e.g. deep learning) for which the model interpretation is difficult. Multimodal models suitable for multi-omic data have been extensively developed to address at least some of these challenges. A wide variety of models have emerged, depending on the use of different statistical methods and theories (e.g., Bayesian or graph) [9], some of them being extensions of well-known machine learning classifiers such as random forests [10].

To reduce omic dimensions, the use of autoencoders (AEs) has progressively been adapted to the integration of multimodal data [11–13]. These neural networks reduce the dimensions of omics data by linearly or non-linearly encoding them into lower-dimensional subspaces which can, in the case of several omics, be concatenated or pre-trained separately before integration [14, 15]. When models are trained separately for each omic and the resulting predictions are later weighted to produce a meta-prediction (or multi-omic prediction), such a design is said to be late integrative. While autoencoding is often used in an unsupervised manner, it is possible to supervise the dimension reduction so that the encoded data is expected to have a higher predictive potential. One problem with AEs is the difficulty in assessing variable importance in reducing dimensions, in contrast to principal component analysis (PCA), where loading factors are easy to consult. Assessing variable importance in semi-supervised autoencoders could, yet, identify variables that are useful for summarizing the data into lower dimensions, but also have high predictive potential. Overall, the benefits of AE approaches are still largely unknown in CVD research, as are the advantages of multi-omics over single-omics approaches for predictive purposes. A key to late-integrative modelling for CVD research is to assess the predictive performance of each encoded omic to be integrated, as well as which ML classifier can best predict CVD or CVD risk factors from these data, and whether supervising AEs allows for better downstream predictions.

**Fig. 1:**
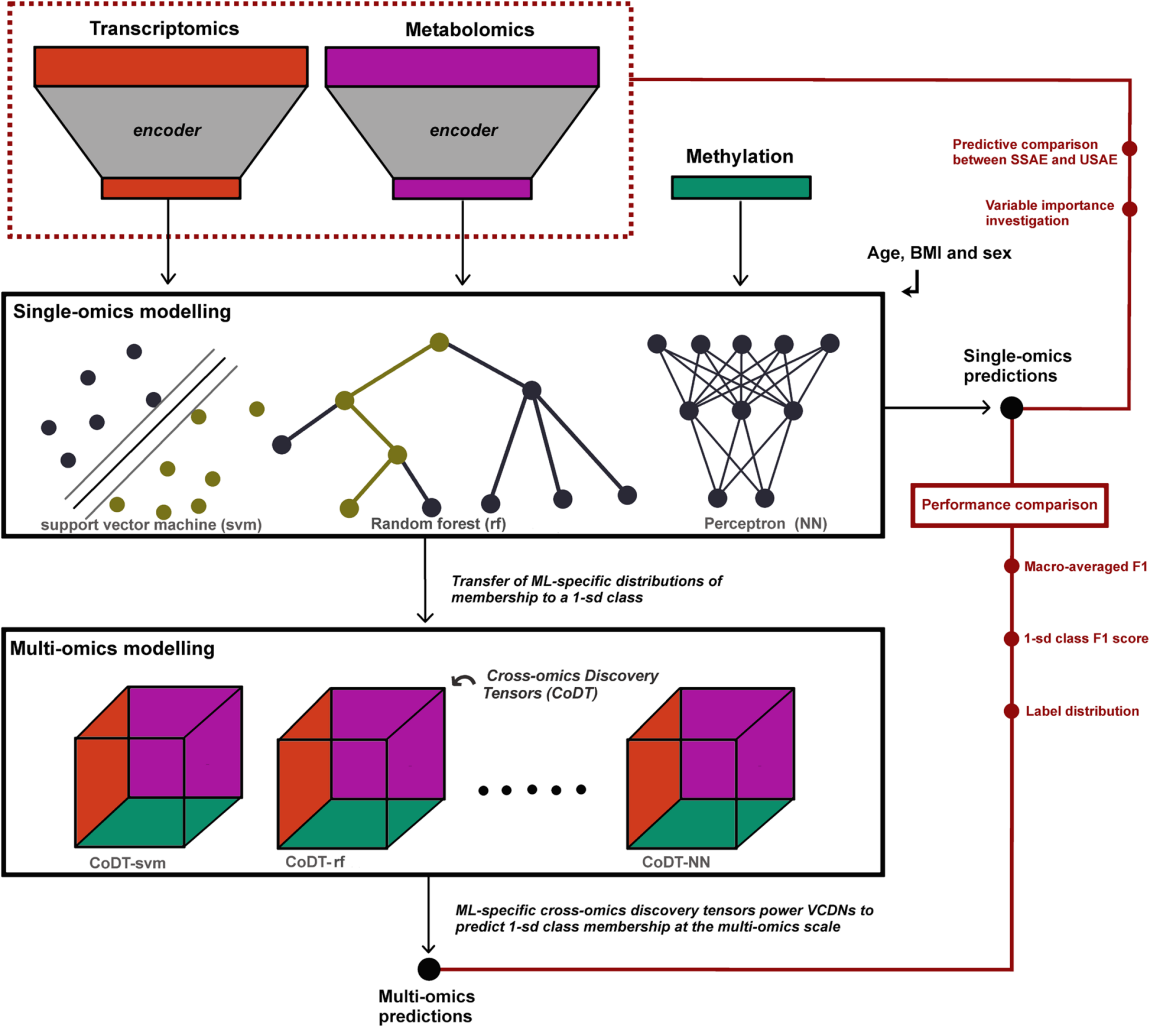
Study pipeline for prediction of individuals with high, low or moderate CVD risk factor values. The study aimed to predict individuals within 3 classes, called 1-sd classes, corresponding to classes of individuals with high, low or moderate CVD risk factor values. CVD risk factors included systolic blood pressure, diastolic blood pressure and three ultrasound-based left ventricular biomarkers. First, the omics data were dimensionally reduced using either pre-filtering or autoencoders, the latter being unsupervised (USAE) or semi-supervised (SSAE). Subsequently, individuals from the test sample were predicted to belong to 1-sd classes at the scale of each encoded omics using different machine learning classifiers. Finally, multi-omic predictions were constructed using a meta-learner: View Correlation Discovery Networks (VCDNs). Multi-omic predictions were constructed by weighting single-omic predictions using VCDNs in the training sample, and predictions in the test sample were then generated

The main goal of our study was to illustrate different machine learning strategies for predicting risk factors under different scenarios, by using CVD risk factors as an example. We sought to investigate the performance of omics data, ML classifiers and autoencoders in predicting individuals with relatively high, low or average CVD biomarker levels, which may reflect individuals potentially at risk and/or protected from CVD (Fig. 1). In addition, we sought to 1) examinate model interpretability in semi-supervised autoencoders to identify which omic factors contributed the most to dimension reduction, 2) investigate in which scenarios late-integrative multi-omic modelling outperforms single-omic modelling in predicting CVD risk factors, and 3) explore whether transfer of omic representations acquired by semi-supervised autoencoders could improve CVD risk factor prediction in an external cohort. To this end, five CVD-related variables were studied: systolic blood pressure (SBP), diastolic blood pressure (DBP), and three biomarkers of left ventricular diastolic dysfunction (LVDD) derived from ultrasound. The joint analysis of blood pressure and LVDD biomarkers was aimed at extending recent multi-omics studies of blood pressure [16, 17], as diastolic dysfunction and development of heart failure are cardiac complications of high blood pressure [18].

## Material and methods

### Cohorts

The Young Finns Study (YFS) is a Finnish prospective initiative that aims to address the multidisciplinary levers underlying cardiovascular disease [19]. From this cohort, different levels of blood-derived omics data, namely transcriptomic, metabolomic, and epigenetic, were collected during 2011 follow-up for up to 1,650 individuals. Participants expressed consent for data collection and use, and the study protocol was conducted according to the guidelines of the Declaration of Helsinki and approved by the relevant ethics committees. Epigenetic DNA methylation data were quantified using Illumina EPIC array, from which pre-processing has been detailed elsewhere [16, 20] and beta values were computed. Transcriptomic data were collected using Illumina microarray technology, and pre-processed [21, 22] resulting in a total of 19,644 initial probes. Metabolomic data were obtained by high-throughput Nuclear Magnetic Resonance (NMR) platform [23, 24]. Four individuals were excluded because three of them had more than 40% missing metabolomic data, and one had inconsistent metabolomic measurements. The metabolomic dataset comprised 228 metabolites and was pre-processed as detailed elsewhere [16]. In addition to the multilevel omics data, age, sex, and BMI were included as covariates.

Two types of CVD biomarkers were studied, namely LVDD biomarkers and blood pressure measurements, as LVDD and high blood pressure are risk factors of CVD [25–29]. The former were measured from medical ultrasound imaging [30, 31] and consisted of the mitral peak velocity of early filling to early diastolic mitral annular velocity (E/e' ratio), the ratio of the early to late ventricular filling velocities (E/A ratio), and Left Atrial Volume Index (LAVI). Systolic and diastolic blood pressure were measured in the sitting position after a 5-min rest using a random zero sphygmomanometer. Only participants for whom the full set of omics data and CVD biomarkers could be overlapped were retained, resulting in a total sample of 1,249 participants with an average age of 42 years (Table 1) of whom 1,000 (~ 80% of the total) were used for model fitting, and the remaining 249 for testing.

**Table 1:** Descriptive characteristics of participants in the training, test and external samples

Cohort	Sample	Size	Variable	Mean (sd)	1-sd classes (X_-1_/X_(-1,+1)_/X_+1_)
YFS	Training	1000	SBP	119 ( 14.2)	15.8/69/15.2 (%)
			DBP	75.2 ( 10.6)	14.5/69.2/16.3 (%)
			E/e’ ratio	4.8 ( 1)	14.4/71.1/14.5 (%)
			E/A ratio	1.5 ( 0.4)	11.7/73.7/14.6 (%)
			LAVI	22.5 ( 6.6)	15.9/69/15.1 (%)
			Age	41.6 ( 5.1)	
			Sex	53.8% (F)	
			BMI	26.4 ( 4.7)	
YFS	Test	249	SBP	119.6 ( 14.1)	14.5/68.7/16.9 (%)
			DBP	75.1 ( 10.6)	16.5/67.1/16.5 (%)
			E/e’ ratio	4.9 ( 1.1)	12.4/67.1/20.5 (%)
			E/A ratio	1.5 ( 0.4)	12.9/72.7/14.5 (%)
			LAVI	22.8 ( 6.4)	12.4/73.5/14.1 (%)
			Age	41.6 ( 5)	
			Sex	53.4% (F)	
			BMI	26.8 ( 5.6)	
FTC	External	310	SBP	151.2 ( 20)	
			DBP	85.8 ( 11.8)	
			Age	62.5 ( 3.8)	
			Sex	58.1% (F)	
			BMI	27.5 ( 4.8)	

Three samples were used in the study; a training sample used during model fitting, a test sample also derived from YFS and an external sample derived from FTC. *YFS* Young Finns Study, *FTC* Finnish Twin Cohort, *sd* standard deviation, *SBP* Systolic blood pressure, *DBP* Diastolic blood pressure, *M* Male, *F* Female. X_-1_: 1-sd class of individuals deviating negatively by at least one sd from the mean. X_+1_: 1-sd class of individuals deviating positively by at least one sd from the mean (i.e., individuals at risk). X_(-1,+1)_: 1-sd class of individuals within 1 sd from the mean

To investigate the value of transferring pre-trained autoencoders to perform a different task within an external cohort, we conducted additional analyses. A total of 310 participants, corresponding to 155 complete twin pairs targeted for blood pressure discordance, were drawn from the elderly subcohort of the Finnish twin cohort (FTC) [32]. This cohort was composed of participants with a mean age of 62.5 years and included a high proportion of hypertensive individuals (Table 1), contrasted with the YFS cohort representative of a 40-year-old Finnish population. The target variables available were averaged systolic and diastolic blood pressure corrected for medication use. The participants were classified as hypertensive if systolic blood pressure exceeded 140 mmHg and if diastolic blood pressure exceeded 90 mmHg; the other participants were considered controls. In this cohort, transcriptomic (Microarray) and metabolomic (NMR) data were also used with independent pre-processing detailed elsewhere [16].

### Single-omics encoding methodology

#### Data processing

The set of target variables consisted of five quantitative measures: SBP, DBP, E/e’ ratio, E/A ratio, and LAVI. During autoencoding, these five variables were adjusted for age, sex, and body mass index (BMI) and used in their residual form; they were kept unchanged otherwise. Such adjustments were made to ensure that the metabolomic and transcriptomic subspaces did not learn a representation dependent on age, sex, or BMI. Target variables were standardized so that an increase of one unit meant a divergence of one standard deviation.

Additional variable filtering was performed for transcriptomic and methylation data, for which the initial number of variables was large. The transcriptomic variables were filtered so that, within the training sample, each selected variable verified one of the following two criteria: 1) the variable was correlated with at least one of the two adjusted blood pressure variables (p-value < 0.05, Pearson correlation nullity test), or 2) the variable was correlated with at least one adjusted LVDD biomarker (p-value < 0.05, Pearson correlation nullity test) and had a variance greater than 0.01. This filtering resulted in a selection of 5,842 probes. Methylation data were filtered by selecting replicated CpG sites from hypertension and CVD literature [33–35], resulting in a set of 75 CpG sites known to be associated with coronary heart disease, myocardial infarction, type-II diabetes, SBP and DBP. The set of metabolomic variables remained unfiltered.

Transcriptomic variables were scaled for model fitting using a *minmax* transformation, defined as *minmax*: u ↦ (u—min_u_)/ (max_u_—min_u_), where min_u_ and max_u_ denoted the minimum and maximum of the variable *u* in the training sample, respectively. Metabolomics data were standardized. The transcriptomic and metabolomic variables in the test sample were scaled from the respective maxima, minima, means, and standard deviation calculated in the training sample. Epigenetic variables were preserved in their beta-value format as no dimension reduction was performed on this omic.

#### Autoencoder architecture and semi-supervision

In order for the AE to learn to extract useful features for representing the omics data, we repeatedly corrupted the input metabolomic and transcriptomic data and used the AE to reconstruct the original data. The corruption consisted of adding Gaussian noise to each variable, with standard deviation 0.01 for the transcriptomic data and 0.1 for the metabolomic data, corresponding to about one-tenth of the mean standard deviations of the transcriptomic and metabolomic variables, respectively.

**Fig. 2:**
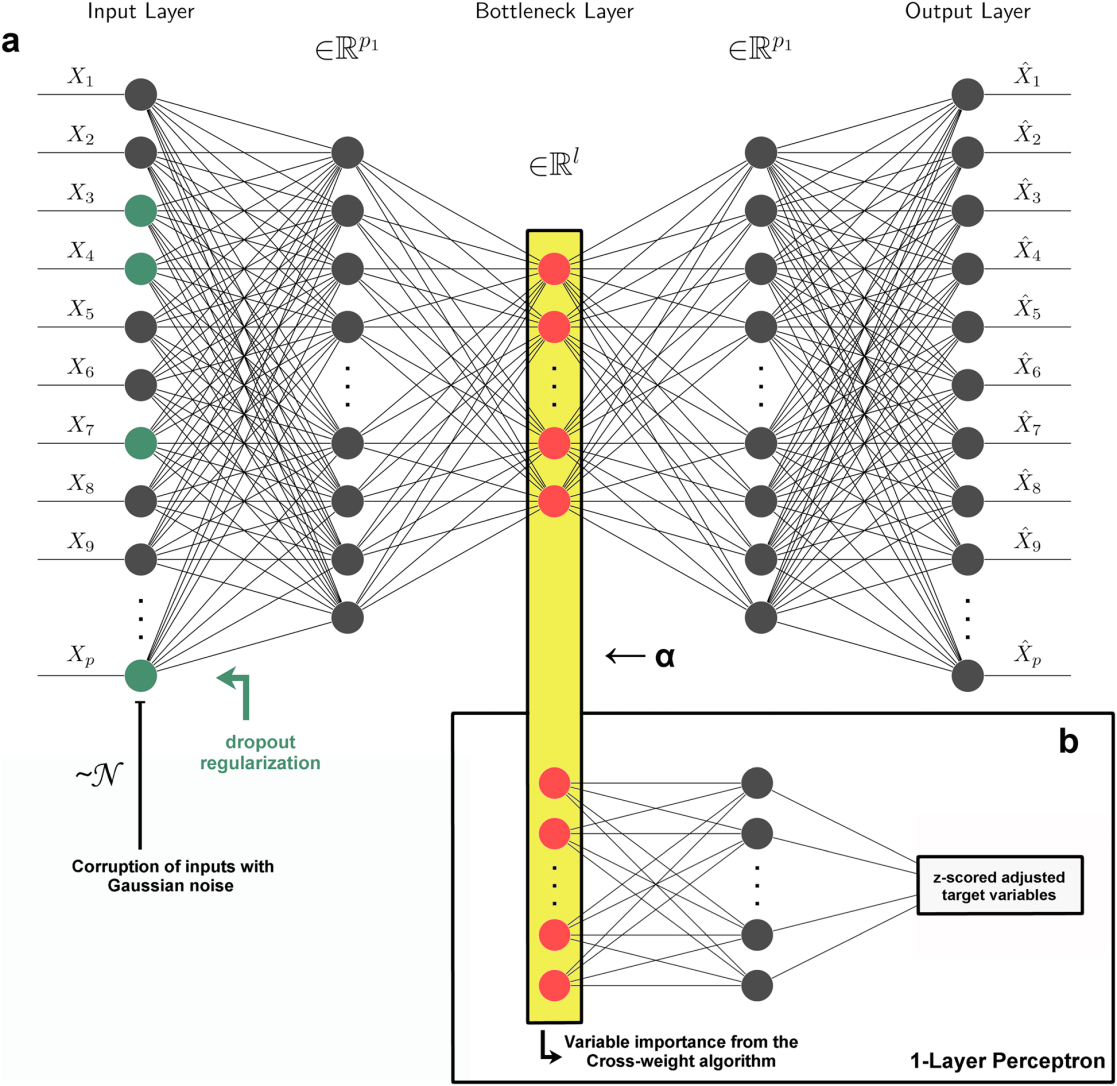
Architecture of the semi-supervised autoencoder. The autoencoder (AE) architecture consisted of an input layer, a hidden layer, and a bottleneck layer corresponding to the subspace layer. A regularization dropout was added on the first layer and the inputs were corrupted with Gaussian noise. To this unsupervised AE (USAE) was grafted a 1-layer perceptron on the bottleneck layer to form the semi-supervised AE (SSAE)

To force the dimension reduction to be optimal for predicting the target variables, we constrained the encoding to learn to predict the target variables in addition to reconstructing the original omics data from the corrupted omics data [36]. In order to perform such a task, we designed a Semi-Supervised Autoencoder (SSAE) which consisted of the junction of a classical AE to which a 1-layer perceptron (1LP) was grafted (Fig. 2). The encoder part was composed of a single hidden layer of dimension p, connected to the bottleneck layer of targeted dimension l. The decoder had a symmetric structure, featuring a hidden layer of dimension p. The 1LP inherited the bottleneck layer and was connected to a hidden layer of the same dimension. The last layer of the decoder and the 1LP were linearly activated to reconstruct the z-scored variables. Other layers were activated with a Leaky Rectified Linear Unit (LeakyReLU) function [37], defined as the identity function if the input *u* is positive, and *au* otherwise where *a* is a real number. This form differs from the uncorrected ReLU [38] activation function (*a* = 0) in that it avoids the dying neuron problem in addition to vanishing gradients that may occur. A dropout regularization was also used on the first layer at a rate *r*. This constraint resulted in a random inactivation of units at a rate *r* and ensured that the input units were not codependent, thus limiting overfitting [39, 40].

In contrast to the use of PCA, there is no unilateral decision rule to determine the size of the autoencoder-derived subspace: its size may depend on multiple parameters (e.g., the number of layers, the number of neurons, etc.). The choice of these parameters is usually guided by the reconstruction quality of the AE, based on metrics such as the Mean Square Error (MSE). While a classical AE focuses mainly on the reconstruction quality of the input, based for example on a metric such as MSE, we wanted SSAE to force the encoding to also take into account the matrix of target variables. We translated this constraint into a bi-output cost function, defined as

1 $${\boldsymbol L}_{\mathbf S\mathbf S\mathbf A\mathbf E}\mathbf{\left({X,\;X^{{}^,},\;Y,\;Y'}\right)}\boldsymbol=\boldsymbol\alpha\boldsymbol L\mathbf{\left({X,\;X^{{}^,}}\right)}\boldsymbol+\mathbf{\left({1-\alpha}\right)}\boldsymbol L\mathbf{\left({Y,\;Y'}\right)}$$

where *L(u, v)* = *∥u − v∥*^*2*^ is a MSE-type loss function, α is a convexity parameter, ***X*** the original data, ***X′*** the reconstructed input, ***Y*** the target matrix composed of z-scored blood pressure measurements and z-scored LVDD biomarkers, and ***Y′*** the reconstruction of ***Y*** from the 1LP. ***L(X, X′)*** and ***L(Y, Y′)*** therefore evaluate respectively the reconstruction quality of the corrupted input into the original data ***X*** and the closeness of the predictions ***Y′*** to ***Y***.

#### Model fitting and evaluation of gains from semi-supervision

We compared the single-omics SSAE model to a corresponding unsupervised model to judge the suitability of our semi-supervised solution for reducing dimensions. This new model, called Unsupervised AE (USAE), had the same neural network structure as the SSAE model, but assigned zero weight to the supervised term of the cost function (i.e. α = 1). USAE can thus be seen as the autoencoder part of SSAE from which the 1LP has been cut out, and it was used to evaluate the value of having the semi-supervising term in the SSAE cost function. To ensure that the encoding performance of USAE was similar to SSAE, we trained USAE using the same configurations. We also ensured that the reconstruction performance of USAE achieved the same reconstruction performance as SSAE, i.e. the same MSE. To do so, we stopped the USAE training phase once the MSE had reached that of the corresponding SSAE model. Thus, the subspaces derived from SSAE and USAE reconstructed the omics input equally well, but the former was expected to be a subspace with stronger predictive potential for the target variables than the latter at an equal reconstruction quality of the omics data.

The tuned hyperparameters were the number of neurons on the hidden layer of the encoder (*nh*), the dropout rate (*r*), the size of the bottleneck layer (*l*), and the convexity parameter (*α*). The values of *r* tested ranged from 0.2 to 0.8 by 0.1, those of *α* from 0.1 to 0.9 by 0.1. Bottleneck layer dimensions were tested starting from 10 by steps of 10 and from 1 by steps of 1 for transcriptomic and metabolomic data, respectively. The number of neurons on the encoder hidden layer was varied from 25 by steps of 25 and from 5 by steps of 5 for transcriptomic and metabolomic data, respectively. Further tests were performed to capture possible performance gains using other activation functions and optimizers. *Adam* and *LeakyReLU*, coupled, showed good training performance; both were therefore kept.

Model fitting was performed using a batch size of 64 and a learning rate of 10^–3^. A large decay rate of 0.9 was chosen, and higher values (0.95 to 0.99) did not show substantial differences in dimension reduction performance from 0.9. One fifth of the training sample was used as a validation sample, consisting of 200 participants. The minimum number of epochs was set to 10 for transcriptomic data and 6 for metabolomic data, and the training procedure was stopped using a moving average of window size of 10 and 6 on the validation cost function, respectively. Therefore, if the validation cost at a given epoch did not improve the average validation cost of the past window epochs, the procedure stopped. The computation was performed with the Keras and Tensorflow modules on the R interface (https://tensorflow.rstudio.com/).

The best model for each omics was considered to be the one with the lowest number of parameters (bottleneck layer size and number of neurons) verifying the following constraints: 1) the encoded subspace reconstructed the input ***X*** from the corrupted input ***X***^***δ***^ with an MSE lower than 0.015 for transcriptomic data and 0.25 for metabolomic data, and 2) the average correlation within the subspace components should not exceed 0.4 for transcriptomic data and 0.3 for metabolomic data. This procedure showed small differences in dimension reduction performance related to the *r* dropout rate on the cost function optimization; a moderate dropout rate *r* = 0.5 was therefore selected for metabolomics and transcriptomics data. The final and optimal architecture of the SSAE encoder consisted of 1) 150 neurons on the hidden layer and a bottleneck layer of size 50 for transcriptomic data, and 2) 55 neurons on the hidden layer and 6 neurons on the bottleneck layer for metabolomic data. The *α* parameter was 0.9 in both cases.

#### Variable importance investigation

**Fig. 3:**
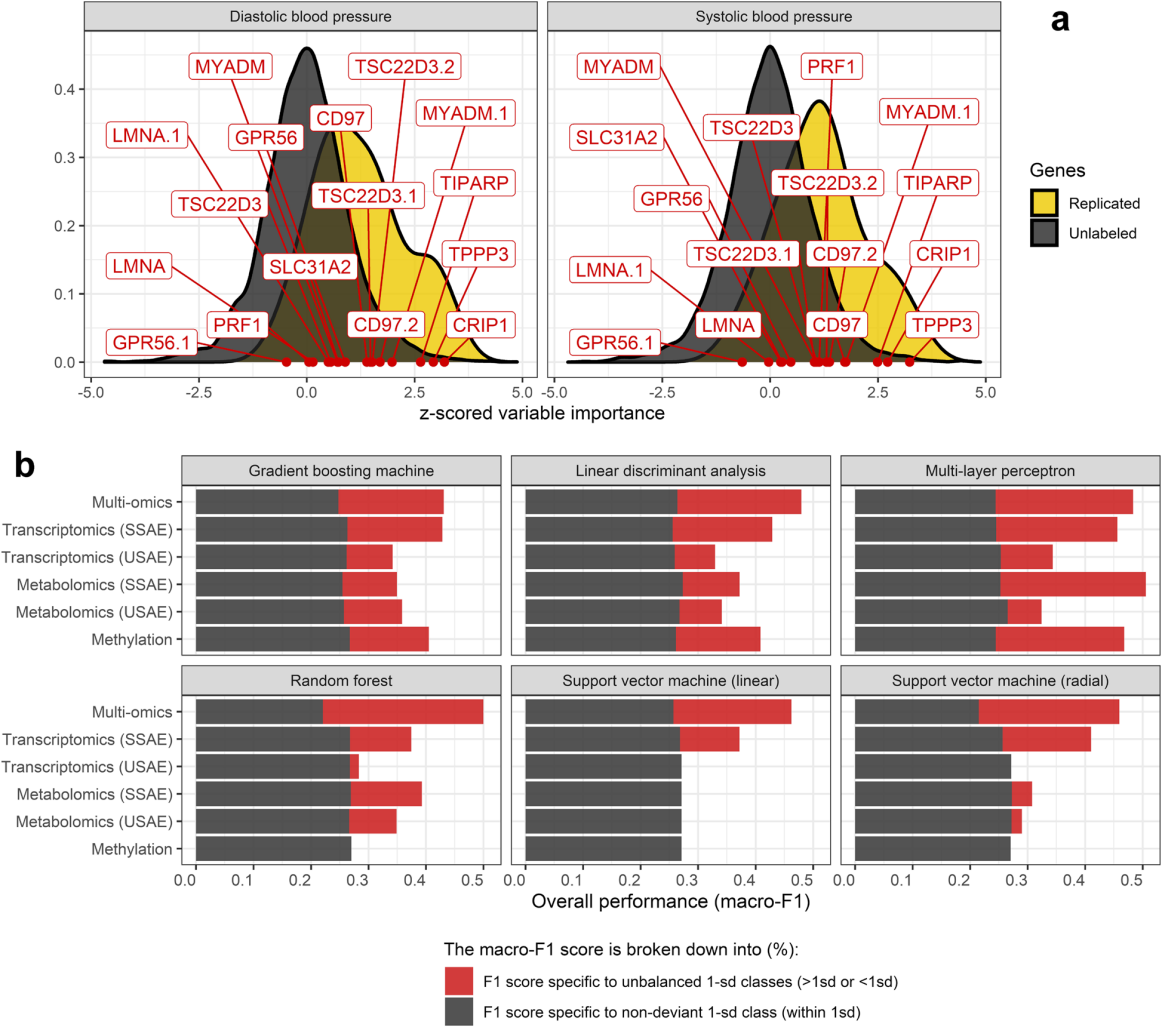
Importance of transcriptomic variables in the reconstruction of blood pressure values and multi-method (cross-)omics performance for systolic blood pressure prediction. In semi-supervised autoencoder dimension reduction, the importance of adjusted variables was quantified using a CW algorithm. The replicated genes in the blood pressure literature have relatively high importance according to the importance values calculated for the other genes in the reconstruction of the adjusted blood variables (a). After the classification of the participants into 1-sd classes, a macro-F1 score, consisting of an unweighted average of 1-sd class specific F1 scores, was obtained for each omics and in a multi-omics configuration, obtained by meta-learning using VCDNs. The multi-omics predictions of the 1-sd classes of systolic blood pressure in the test sample were better compared to those obtained in single-omics configurations for 5 of the 6 classifiers used (b). This superiority was illustrated in particular in the ability of the multi-omics modelling to acquire good predictive performances within the imbalanced 1-sd classes, as shown with the support vector machines. The performances presented in b) are also available for the other target variables in the supplementary material. CW: Connection Weight; VCDN: View Correlation Discovery Networks

There are a variety of methods for estimating the importance of input variables in predicting an outcome within a neural network, but none is considered gold standard. We propose to use one of these methods, called the Connection Weights (CW) algorithm, to estimate the importance of omics variables in SSAE modelling (Fig. 3a). This method, commonly referred to as the Olden method, computes the product of weights across layers of the neural network and has proven to be a reliable method for estimating variable importance [41]. This approach contrasts with Garson's method in that the sign of the variables' contributions are preserved in addition to their magnitude, and it is possible to adapt the CW algorithm to several layers [41].

In our SSAE modelling, the CW algorithm involved the weights of the encoder and 1LP layers. The importance of an input variable ***x***_***i***_ (*i* ∈ ℕ) in reconstructing a continuous target outcome variable ***y***_***j***_ (*j* ∈ {1, 2, 3, 4, 5}) was referred to as ***RIx***_***i***_***(y***_***j***_***).***

### Single-, inter- and multi-omics predictive methodology

#### Classification of participants into 1-sd classes

The YFS cohort is composed of relatively young participants and is expected to represent the general CVD risk profile of the Finnish population in that age group. The unadjusted blood pressure variables and biomarkers of LVDD were standardized, and each variable was classified into 3 classes (Table 1). The 3 classes were defined according to the participants' distance from the training sample mean, with 1 standard deviation (sd) as the distance criterion. The three 1-standard deviation classes (1-sd classes) created for each target variable thus consisted of 1) participants deviating negatively by at least 1 sd from the mean, 2) participants deviating positively by at least 1 sd from the mean, and 3) participants within 1 sd from the mean.

#### Predictive performance of omic subspaces

To evaluate the predictive performance of each omic subspace compared to the other omic subspaces but also to the unsupervised homologous subspace (via USAE), a panel of machine learning (ML) classifiers was used to predict 1-sd class membership from the information on the bottleneck layer. These methods included random forests, linear and nonlinear support vector machine (svm), linear discriminant analysis, a gradient boosting machine (GBM) model and a Multi-Layer Perceptron (1 hidden layer). Each omic subspace was scaled and age, sex, and BMI were added for model training. The ML classifiers were all trained using the caret R package [42]. A five fold cross-validation was used during model fitting within the training sample to select the best performing model configurations. The 1-sd class membership of each target variable was then predicted for the 249 participants in the test sample.

Due to the imbalanced design (Table 1), we used an F1 score, defined as the harmonic mean between precision and recall, where *precision* = *TP/(TP* + *FP)* and *recall* = *TP/(TP* + *FN)*, with *TP* the number of true positives, *FP* the number of false negatives and *FN* the number of false negatives. The value of using an F1 score also lay in its ability to assign a specific F1 score to each class, which allowed us to observe which omics data best predicted a specific 1-sd class. A macro-averaged F1 score, defined as an unweighted average of the three 1-sd class-specific F1 scores, was then used to describe the overall performance of each ML classifier. The use of the unweighted macro-averaged F1 score thus rewarded the ML models for predicting relatively well also the risk and protected 1-sd classes for which the numbers were smaller than the 1-sd class of participants close to the population mean.

#### Cross-omics integration for multi-omics prediction

An integrative multi-omics modelling aiming at combining single-omic subspace predictions (i.e. label spaces) was used to predict the 1-sd classes. For this purpose, we used View Correlation Discovery Networks (VCDNs) [43, 44]. Briefly, VCDNs exploit 1) inter-omics correlations in the label space for classification tasks, and 2) the fact that some omics might better predict a 1-sd class, which incidentally can be assessed from F1 scores.

From a technical point of view, VCDN was defined as a fully connected neural network taking as input a rescaled Cross-omics Discovery Tensor (CoDT), as introduced elsewhere [44]. We built CoDTs from the 1-sd class membership probabilities derived from each omic subspace and each ML classifier, for each target variable (Fig. 1). In this way, we derived for each omic subspace *o*_*i*_ (*i* = 1, 2, 3) and for each individual *k* a ML classifier-specific vector ***ŷ***_***k***_***(o***_***i***_***)*** ∈ [0,1]^*c*^ having *c* = *3* probability entries corresponding to the probabilities of belonging to the 1-sd classes of the considered target variable. Next, a CoDT was constructed such that each entry *e*_*i*_ was defined as a product of three probabilities *e*_*i*_ = *abc*, where {*a*,*b*,*c*} was a combination of 1-sd class probabilities across the 3 omics. Thus, not only were the probabilities of belonging to the same classes subject to multiplication, but also the probabilities of belonging to different classes from different omics. The total number of combinations was therefore 3^3^ = 27, corresponding de facto to the input size of the VCDN, to which a hidden layer of dimension *c*^*2*^ and an output layer of size *c* were added. For each target variable and each classifier, the layers were activated with *LeakyRelU* and VCDNs were trained under *Adam* optimizer. Once the VCDNs were trained, the label vectors of the test participants were predicted. The F1 score was used to evaluate the quality of the multi-omics predictions and compared to those obtained in single-omics settings.

#### Transfer learning to the external cohort

We predicted hypertensive individuals in the FTC by transferring the learning acquired from semi-supervised autoencoders trained on the YFS cohort (Fig. 5a). The modelling consisted of building a neural network inheriting the layers of the SSAE’s encoder part pre-trained on the YFS, to which a task-specific hidden layer (Fig. 5b), a dropout regularization, and three clinical variables (sex, age, and BMI) were added (Fig. 5a). A proportion of FTC participants was used for fine-tuning, having the function of adjusting the weights of the new, non-transferred layers; the remaining participants were used to assess the predictive performance of the model, measured from the AUC derived from the prediction of hypertensive status. The AUC was calculated in participants in the FTC test subset by randomly distributing 20%, 40%, 60%, and 80% of the FTC participants for fine-tuning. The procedure was repeated 100 times. A homologous clone model was created from scratch for comparison with the model based on transfer learning. This clone model had the same structure as the model based on transfer learning, but did not inherit the initial weights; the first layers were trained using transcriptomic or metabolomic data. In both cases, model fitting was performed under the same conditions.

## Results

Briefly, we intended to predict individuals deviating positively or negatively from at least one sd of the population mean or being within 1sd from the mean, for five risk factor outcomes (Fig. 1). This led to three classes for each outcome, which we refer to as 1-sd classes. The membership of these classes was predicted in a test sample, and we compared the quality of predictions as a function of the ML classifier used, the encoded omics used, and whether the autoencoding was semi-supervised or not. In addition, we investigated variable importance in semi-supervised autoencoding of metabolomic and transcriptomic data. Multi-omic predictions were constructed using a meta-learner and compared to single-omic predictions, for each ML classifier. Finally, we transferred the omics representations learned by SSAE in the YFS cohort to another external cohort to investigate whether this would improve the identification of hypertensive participants compared to modeling from scratch.

### Variable importance in semi-supervised encoding

We investigated the importance of the metabolomic and transcriptomic variables in reconstructing the CVD biomarkers values adjusted by sex, BMI and age (Fig. 2). To perform this task, the CW algorithm computed a cross-product of the weights defined through the layers of the encoder and the 1LP grafted to the bottleneck layer.

The metabolomic features of highest absolute variable importance in the reconstruction of LVDD biomarker values were mainly lipids, cholesterol concentrations, lactate and citrate (Table 2). Branched-chain amino acids (BCAAs) were also prominent, both in reconstructing adjusted LVDD biomarker values and adjusted systolic blood pressure measurements. Fatty acids were also found to be variables of high importance. Metabolomic variables of highest ordered importance can be found in Table 2.

**Table 2:** Metabolomic variables with the highest absolute variable importance in the reconstruction of adjusted left ventricular dysfunction biomarker values and adjusted blood pressure values in semi-supervised autoencoding

Target	Variable importance (z-scored ***RI***)
SBP	Citrate (3.48); Free cholesterol to total lipids ratio in very small VLDL (-2.94); Total lipids in small HDL (2.83); Creatinine (-2.83); Acetate (-2.64); Glycine (-2.54); Saturated fatty acids (2.51); Cholesterol esters to total lipids ratio in very small VLDL (2.43); Valine (-2.37); Ratio of saturated fatty acids to total fatty acids (2.27)
DBP	Lactate (3.65); Free cholesterol to total lipids ratio in very small VLDL (-3.51); Citrate (3.26); Creatinine (-2.59); Glycine (-2.43); Saturated fatty acids (2.26); Total lipids in small HDL (2.2); Pyruvate (2.15); Total cholesterol to total lipids ratio in medium VLDL (-2.15); Phospholipids in small HDL (2.07)
E/A ratio	Lactate (-5.75); Pyruvate (-3.74); Creatinine (3.26); Citrate (-2.87); 3-hydroxybutyrate (-2.63); Omega-3 fatty acids (2.51); Free cholesterol to total lipids ratio in very small VLDL (2.36); Isoleucine (2.05); Cholesterol esters to total lipids ratio in very large HDL (1.92); Estimated degree of unsaturation (1.85)
E/e’ ratio	Triglyceride Cholesterol esters to total lipids ratio in very small VLDL (-3.43); Citrate (-3.23); Total cholesterol to total lipids ratio in very small VLDL (-2.94); Total lipids ratio in small VLDL (2.41); Cholesterol esters in very small VLDL (-2.36); Pyruvate (2.35); Albumin (2.33); Leucine (2.24); Glucose (2.21); Valine (2.2)
LAVI	Lactate (-6.00); Pyruvate (-4.36); Free cholesterol to total lipids ratio in small VLDL (2.57); Acetate (-2.55); Omega-3 fatty acids (2.42); Free cholesterol to total lipids ratio in very small VLDL (2.08); Free cholesterol to total lipids ratio in medium VLDL (2.06); Free cholesterol to total lipids ratio in small HDL (2); Creatinine (1.96); Cholesterol esters in small VLDL (1.94)

The most important metabolomic variables in the reconstruction of adjusted CVD biomarker values included lipids, cholesterol concentrations but also branched-chain amino acids, citrate and lactate. RI: Variable importance score obtained using Connection Weight algorithm. *SBP* Systolic blood pressure, *DBP* Diastolic blood pressure. E/e’ ratio: mitral peak velocity of early filling to early diastolic mitral annular velocity. E/A ratio: ratio of the early to late ventricular filling velocities, *LAVI* Left Atrial Volume Index, *HDL* High-density lipoprotein, *VLDL* Very-low-density lipoprotein

For transcriptomic data, genes reported in the literature as being associated with systolic and diastolic blood pressure were further studied [45, 46], as 16 probes could be found among the 5,842 transcriptomic variables. These reported genes included *TPPP3*, *CRIP1*, and *TIPARP* which were in the first and/or second percentile of genes with the greatest absolute variable importance values in the reconstruction of adjusted systolic and diastolic blood pressure values. The mean absolute importance score of the reported transcriptomic variables corresponded to the 15th and 16th percentiles of the greatest absolute importance scores of SBP and DBP, respectively (Fig. 3a).

### Single- and multi-omics predictive performance

Disparities in performance were observed depending on the ML classifiers applied to the encoded representations of omics data. The best classifiers at single-omics levels were the Multi-layer perceptron (MLP) and the GBM, for which the macro-F1 scores were the highest (Table 3). The linear and non-linear svm showed poor performance in predicting imbalanced classes of LVDD biomarkers (Supplementary material: Figure S1, S2, S3 and S4) as macro-F1 scores in the test subset were most often close to a naive classifier (naive classifier macro-F1 = 33.3% with a 15%/70%/15% design (Table 1)).

**Table 3:** Performance of the best single-omics and multi-omics machine learning model for each target variable in the test subset

Target	Model	Omics	F1(X_-1_)	F1(X_(-1,+1)_)	F1(X_+1_)	macro-F1
DBP	MLP	Transcriptomics	*.*22	*.*75	*.*42	*.*47
DBP	MLP	Metabolomics	*.*16	*.*74	*.*51	*.*47
DBP	GBM	Methylation	*.*12	*.*79	*.*32	*.*41
DBP	rf	Multi-omics	***.*36**	*.*68	***.*55**	***.*53**
SBP	MLP	Transcriptomics	*.*25	*.*74	*.*38	*.*46
SBP	MLP	Metabolomics	*.*35	*.*76	*.*41	***.*51**
SBP	MLP	Methylation	*.*31	*.*73	*.*36	*.*47
SBP	rf	Multi-omics	**.40**	.66	**.44**	.50
E/A ratio	GBM	Transcriptomics	***.*27**	*.*83	*.*23	***.*44**
E/A ratio	GBM	Metabolomics	*.*05	*.*83	*.*26	*.*38
E/A ratio	MLP	Methylation	*.*10	*.*82	*.*20	*.*37
E/A ratio	MLP	Multi-omics	*.*05	*.*81	***.*28**	*.*38
E/e’ ratio	GBM	Transcriptomics	*.*14	*.*78	*.*27	*.*40
E/e’ ratio	GBM	Metabolomics	***.*32**	*.*77	*.*07	*.*39
E/e’ ratio	MLP	Methylation	*.*19	*.*74	*.*15	*.*36
E/e’ ratio	rf	Multi-omics	*.*29	*.*68	***.*32**	***.*43**
LAVI	GBM	Transcriptomics	*.*05	*.*82	*.*10	*.*32
LAVI	GBM	Metabolomics	*.*10	*.*81	*.*04	*.*32
LAVI	MLP	Methylation	*.*04	*.*81	*.*14	*.*33
LAVI	rf	Multi-omics	***.*31**	*.*47	***.*25**	***.*34**

ML classifier performance comparisons were based only on omics dimensionally reduced with semi-supervised autoencoders, except for the epigenetics domain where feature selection was performed instead. The blood pressure variables were the best predicted in the test sample. For each target variable, the best 1-sd class-specific predictions for less represented classes and macro-F1 scores are highlighted in bold. F1(X_-1_): F1 score of the 1-sd class of individuals deviating negatively by at least one sd from the mean. F1(X_+1_): F1 score of the 1-sd class of individuals deviating positively by at least one sd from the mean (i.e., individuals at risk). F1(X_(-1,+1)_): F1 score of the 1-sd class of individuals within 1 sd from the mean. *SBP* Systolic blood pressure, *DBP* Diastolic blood pressure. E/e’ ratio: mitral peak velocity of early filling to early diastolic mitral annular velocity. E/A ratio: ratio of the early to late ventricular filling velocities. *LAVI* Left Atrial Volume Index, *sd* standard deviation, *GBM* Gradient Boosting Machine, *MLP* Multi-layer Perceptron, *rf* random forest

Predictions derived from metabolomic and transcriptomic semi-supervised subspaces in the test subsample were at least as good as those obtained in unsupervised configurations in 76% of cases, all methods and target variables combined. Macro-F1 scores obtained in a semi-supervised setting were strictly better than those obtained in an unsupervised setting for transcriptomic data in 70% of the cases. The semi-supervised transcriptomic subspaces strictly improved predictions of individuals deviating more than 1 sd from the mean in 73% and 60% of the cases, respectively. Macro-F1 scores of predictions derived from SSAE-trained metabolomic subspaces outperformed USAE metabolomic predictions in half of the cases, and predictions were equal in a quarter of the cases. Predictions of individuals above or below 1 standard deviation from the mean were similar 40% and 46% of the time, respectively. However, when predictions were not equal, SSAE-trained metabolomic predictions outperformed USAE metabolomic predictions more than two-thirds of the time.

**Fig. 4:**
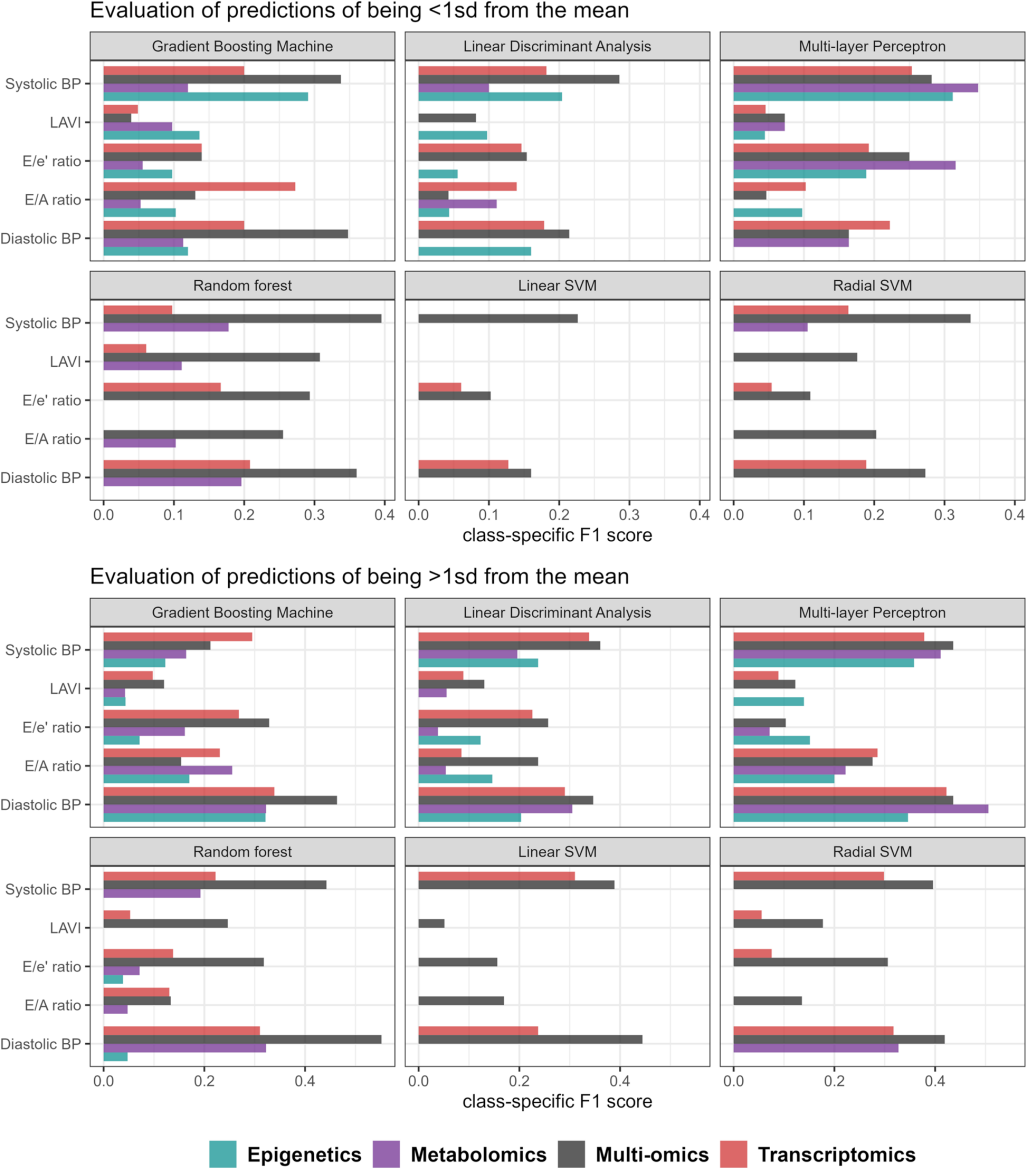
Performance of machine learning classifiers in predicting less represented classes from omics data in the test sample. 1-sd class F1 scores for predicting individuals above or below 1 standard deviation of the mean are shown for each machine learning classifier and omic data. ML classifiers were applied to dimension-reduced omic data from semi-supervised autoencoders, except for the epigenetics domain where feature selection was performed instead. Multi-omic predictions were constructed using a meta-learner for each ML classifier and CVD risk factor independently. Diastolic BP: Diastolic blood pressure. Systolic BP: Systolic blood pressure. E/e’ ratio: mitral peak velocity of early filling to early diastolic mitral annular velocity. E/A ratio: ratio of the early to late ventricular filling velocities. LAVI: Left Atrial Volume Index. sd: standard deviation

Semi-supervision of the omic subspaces using SSAE thus, across all ML methods and target variables, improved predictive performance, notably by better predicting the imbalanced 1-sd classes. More information specific to each ML method or CVD biomarker can be found in Supplementary material, Fig. 3b and Fig. 4.

Multi-omics predictions derived from meta-learners (see Methods) outperformed single-omic predictive modelling in predicting 1-sd classes of blood pressure 83% of the time; only MLPs showed no superiority of the multi-omics approach over those acquired in single-omics configurations (Fig. 3b; Supplementary material: Figure S1, S2, S3 and S4). Among all classifiers, random forest had the best performance for multi-omics modelling (Table 3). At the global scale, the multi-omics approach obtained the best macro-F1 scores in the test subsample except for SBP and E/A ratio, for which the metabolomics data (macro-F1 of 0.51 vs. 0.50 for multi-omics) and the transcriptomics data (macro-F1 of 0.44 vs. 0.38 for multi-omics) provided the best predictions, respectively. Interestingly, among the best classifiers of each target variable, the multi-omics approach obtained the worst predictions of the non-deviant 1-sd class individuals (within 1 sd of the mean) (Fig. 3b; Supplementary material: Figure S1, S2, S3 and S4) but best predicted individuals deviating by more than 1 sd from the mean (Fig. 4; Table 3).

As the multi-omics predictions (with random forest classifier) for membership in the 1-sd classes of blood pressure were found to be the best (Table 3), we investigated the potential of using these predictions to explain the variation in blood pressure values. Therefore, we fitted univariate linear regressions and examined the coefficients of determination R^2^. The predicted probabilities of having a blood pressure greater than 1 sd of the mean explained, alone, 13.6% and 21.4% of the systolic and diastolic blood pressure variation in the test subset, respectively. The addition of the predictions of belonging to the class of individuals deviating negatively from 1 sd of the mean and of the three a priori clinical variables (age, sex, and BMI) ultimately explained 27.3% and 32.0% of the variation in SBP and DBP in the test subset, respectively.

### Transfer learning

**Fig. 5:**
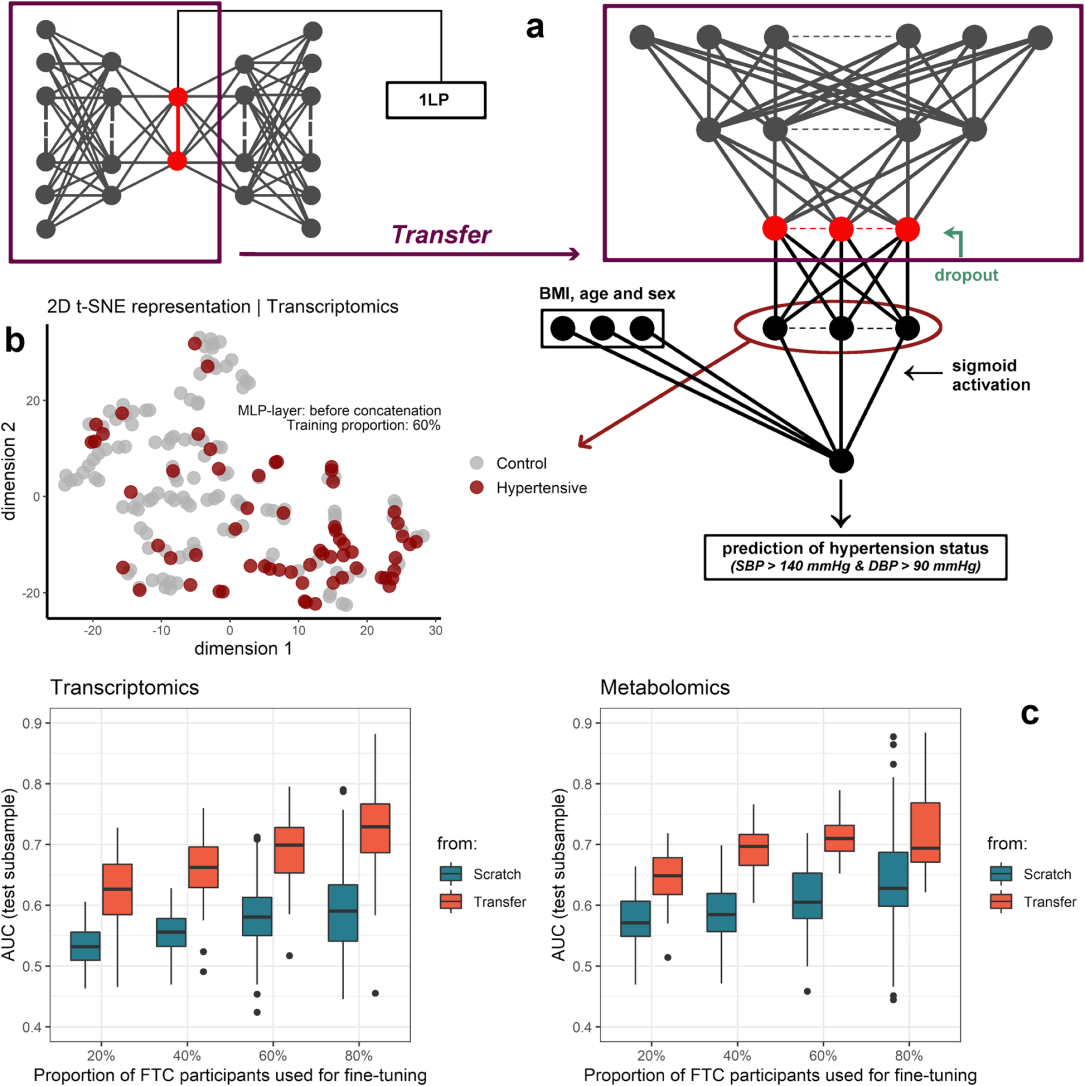
Replication by transfer learning for detection of hypertensive participants in the external Finnish Twin Cohort. The weights of the SSAE encoding layers pre-trained distinctly on the YFS metabolomic and transcriptomic data were transferred for the detection of FTC hypertensive individuals (a). To this pre-trained structure, a dropout regularization was added, and an additional layer with the function of learning a task-specific representation was added (b) and concatenated to three clinical variables: age, sex, and BMI. For metabolomic and transcriptomic data, the constructed neural network was refined by randomly selecting 20%, 40%, 60%, and 80% of the FTC sample. This was repeated 100 times, the AUC in the remaining test subsamples was calculated and compared to a cloned model not inheriting the pre-trained weights on the YFS data (c), i.e. having random weights and learning its task from scratch. SSAE: semi-supervised autoencoder. YFS: Young Finns Study. FTC: Finnish Twin Cohort. BMI: body mass index. AUC: area under the curve

One of the major challenges of multi-omics approaches is the externalization of models to cohorts 1) composed of individuals clinically different from those used during the training phase, 2) for which omics preprocessing and instrumentation were performed independently or were different from those used during model fitting, resulting in batch effects, and 3) for which the predictive objectives may differ. A solution to these issues can be found in transfer learning. We transferred the learning acquired in the YFS cohort (Fig. 5a), i.e., the pre-trained weights of the SSAE encoder layers, to predict hypertensive participants in an external cohort (Table 1) using unequal proportions of FTC participants to fine-tune the model (see Methods section).

Regardless of whether 20%, 40%, 60%, or 80% of the FTC sample was taken for fine-tuning, the model with transfer-inherited first layers outperformed the one constructed from scratch for both transcriptomic and metabolomic data (Fig. 5c). This superiority was reflected in a median gain in Area Under the Curve (AUC) in the test subsample ranging from 0.09 to 0.14 and 0.07 to 0.11 units for transcriptomic and metabolomic data, respectively, across all fine-tuning combinations. The median AUC obtained by transfer learning increased from 62.7% to 72.9% (*resp.* 53.2% to 59.0% from scratch) and from 64.9% to 69.4% (*resp.* 57.1% to 62.8% from scratch) by increasing the fine-tuning sample size from 20 to 80% for transcriptomic and metabolomic data, respectively. Increasing the fine-tuning sample therefore increased the median AUC from poor to moderate using transfer learning, and from very poor to poor when modelling from scratch.

The increase in median AUC as a function of fine-tuning subsample size was, however, 75% greater in the transfer-based model than in the model built from scratch for transcriptomic data, whereas no substantial increase in difference was observed for metabolomic data. Transcriptomic data also best predicted hypertensive status of participants in the test subset when 248 participants (i.e., 80% of the FTC set) from the FTC cohort were used for fine-tuning (median AUC: 72.9%), although performing worse than metabolomic data for small fine-tuning subsample sizes (Fig. 5c). The transfer has therefore mostly benefited transcriptomic data for which the high dimensions are a hindrance to modelling from scratch.

## Discussion

Our study aimed to illustrate different machine learning strategies for predicting risk factors, which we compared under different scenarios, with CVD as a real-world example. Although we used a unique training dataset, suggesting that generalization of our results should be made with caution, it illustrates how the choice of autoencoders, ML classifiers, or omics data influences downstream predictions of CVD risk factors, and therefore provides a valuable platform for the scientific community. We showed that multi-omics modelling outperformed single-omics modelling particularly in predicting individuals in less-represented classes, being classes of interest for the detection of individuals at risk of CVD. Compared to their classical unsupervised counterparts, semi-supervised autoencoders allowed for better downstream predictions. They also allowed quantifying the importance of transcriptomic and metabolomic variables in reconstructing adjusted cardiovascular disease biomarkers. In addition, transfer learning to an older and smaller cohort with a high prevalence of hypertensives led to major performance gains, showing the replicative potential of pre-trained semi-supervised autoencoders.

While multi-omics predictions outperformed single-omics predictions on average, very few scenarios showed little to no improvement in prediction. This echoes known results from cancer research [47], which we also observed for CVD risk factors. This highlights the need to consider the costs and benefits of adding omics layers, as adding data does not systematically improve model performance but increases model complexity in the training set, which could reduce model reproducibility in external cohorts. For example, our study suggests that plasma metabolites are highly predictive of systolic blood pressure for which the addition of other omics layers did not substantially improve prediction. This is consistent with multi-omic studies of blood pressure where metabolomic data were found to be the best predictor of systolic blood pressure as well [16, 17]. However, our study suggests that the predictive potential of metabolomic data compared to other omics varies depending on which ML classifiers are used.

Metabolomic variables playing a substantial role in the reconstruction of CVD biomarkers included lipids and cholesterol concentrations (Table 2), for which associations with CVD risk are now well established [48, 49]. The presence of lipid and cholesterol concentrations as well as BCAAs among the variables of high importance in the reconstruction of adjusted blood pressure values echoes previous results observed in a multi-omics study of blood pressure [16], as does the presence of saturated fatty acids, glycine, lipids, BCAAs and lactate with respect to another recent multi-omics study of blood pressure [17]. Metabolites of high importance in reconstructing LVDD biomarkers included fatty acids [50] and BCAAs [51], reflecting recent findings in left ventricular function. Results were therefore consistent with the literature, making semi-supervision an interesting explanatory tool, even when semi-supervision could not provide a better 1-sd class predictive performance. However, because our study does not involve statistical testing but rather illustrates the use of variable importance examination, the generalizability of these results to epidemiologic studies is limited.

The interpretability of semi-supervised autoencoders faces major obstacles in genomics. The first is methodological: the interpretability of advanced ML models remains an active research area [52] for which a substantial number of methods have only recently emerged in genomics. Various methods exist for assessing variable importance, such as SHAP values, but little is known about the best strategy for assessing variable importance when using omics data. The second obstacle mirrors the first, as the high omics dimensions can slow down the discovery potential, as in the case of transcriptomic data for which the false discovery rate could not be controlled downstream in our framework, thus limiting their potential for interpretation. High input correlations could also influence measures of variable importance. The last obstacle is aetiological, as causal inference is not possible in such a setting, which significantly limits the biological dimension in the use of autoencoders. Thus, estimates of variable importance are not necessarily an indication of biological importance. From an epidemiological perspective, using methods adapted to causal inference is therefore preferable; mendelian randomization in this context is largely appropriate [53]. The biological nature of the omics presents its own challenges, as transcriptomics and methylation data reflect the activity of the cells from the tissue that has been sampled, while metabolomics represent the flux of molecules from varied sources. Obtaining the relevant target tissue in large numbers is a major challenge, ethically and logistically. Model organism studies and in vitro cell studies could provide more insight to discoveries from multi-omics modelling on observational data.

In addition to the difficulties in modelling omics data due to their biological complexity, the CVD risk factors of the current study are also subject to biological limitations. Consecutive systolic or diastolic blood pressure measurements are not perfectly aligned, making blood pressure measurement highly variable. For example, we observed correlations ranging 0.73–0.92 and 0.76–0.90 between four SBP and DBP measurements taken on the same morning in the FTC sample, respectively. This short-term variability in blood pressure measurements can introduce noise into the variables, thereby limiting the predictive potential of any statistical model, whether advanced or not. To partially address the noise in blood pressure measurements, we have identified two possible alternatives to blood pressure. First, we suggest increased use of polygenic risk scores and the underlying genetic components of blood pressure [54], because they are not influenced by short-term changes in time. Second, we recommend that more use be made of nighttime blood pressure measurements. These may be, when available, less prone to variability as confounding external factors (e.g., stress, white coat effect) may be reduced. Nighttime blood pressure measurements may also provide a better estimate of a patient's health status, as they have been shown to be better predictors of incident cardiovascular disease than traditional daytime measurements [55]. Finally, it should be noted that CVD risk factors in our study were categorized and not used as continuous variables. We used categorized outcomes to illustrate and mimic the use of different machine learning strategies in clinical settings, as blood pressure and LVD outcomes are based on thresholds in practice. However, categorization of CVD risk factors may result in a loss of information contained in the variables.

Another obstacle in model-reproducibility was observed with the partition of YFS individuals into classes based on LVDD biomarkers values. In contrast to systolic and diastolic blood pressure for which unitary increases are causally associated with the occurrence of cardiovascular disease [26, 56], and for which positive deviation from the mean implies a higher risk of developing CVD, knowledge of LVDD biomarkers is so far mainly based on thresholds. Thus, deviation from the mean in a non-selected cohort of relatively healthy individuals such as the Young Finns Study may not necessarily imply an increased risk of developing CVD. This design limitation was, moreover, reflected in the quality of LVDD biomarker predictions, which were relatively weak compared with SBP and DBP predictions; our ML classifiers had difficulty distinguishing among relatively healthy individuals. The use of a cohort with a substantial proportion of individuals at high risk for CVD therefore seems, in the context of a study focusing on diastolic function, more appropriate. Finally, despite better predictions of blood pressure outcomes from ML classifiers, these may, at least in part, reflect the prediction of poor or good overall health rather than blood pressure itself. As discussed previously, the use of advanced machine learning is not systematically suited to epidemiologic perspectives because confounders are not easily adjusted in such settings. Measures of ML classifier performance are therefore expected to be affected by confounding [57].

As for the challenges associated with omics data integration, we chose to dissociate single-omics views and multi-omics views. This choice was in line with the aim of our study, which was to illustrate and compare different machine learning strategies for predicting CVD risk factors in different scenarios, including the separate study of omics. One could use an end-to-end and single-task setting (Fig. 1), limiting the study to a single target variable by joining classification, dimension reduction and meta-learning. However, the aim of our study was to present and explore different machine learning strategies, which we applied to the prediction of CVD risk factors. To this end, the use of methods specifically designed for multi-omic data integration is preferable, to which our study complements, as we have shown, for example, benefits in supervising dimension reduction and transfer learning. The use of larger cohorts, for example from large biobanks, could not only improve ML classifier performance but also the potential for outsourcing models to external cohorts, as could greater Gaussian corruption of neural inputs or feature selection based on a priori knowledge. The use of large biobank datasets would also allow for greater generalizability of results, which is a limitation of our study. The further development of multi-omics methods, within larger cohorts and in multiple settings, is therefore a promising approach for studying cardiovascular disease risk factors within a machine learning framework.

## Conclusions

Our study comprehensively illustrates the use of different machine learning strategies in predicting risk factors under different scenarios, using CVD as an example. In particular, we demonstrate the advantages of using supervised autoencoders and transfer learning in the study of CVD risk factors, as well as the influence that the choice of omics and ML classifiers can have on the quality of predictions. We therefore believe that the present study can provide an excellent platform for CVD researchers, but also for a broader audience interested in the use of omics data.

### Supplementary Information

**Supplementary Material 1.**

## Data Availability

YFS data supporting the conclusions of this article were obtained from the Cardiovascular Risk In Young Finns study (YFS) after submission and approval of our study plan by the YFS coordinators. The YFS dataset comprises health related participant data and their use is therefore restricted under the regulations on professional secrecy (Act on the Openness of Government Activities, 612/1999) and on sensitive personal data (Personal Data Act, 523/1999, implementing the EU data protection directive 95/46/EC). Due to these legal restrictions, the data from this study can not be stored in public repositories or otherwise made publicly available. However, access to the data can be granted on a case-by-case basis, upon request, by contacting the corresponding author. Data sharing outside the group is done in collaboration with YFS group and requires a data-sharing agreement. Investigators can submit an expression of interest to the chairman of the publication committee (Prof Mika Kähönen, Tampere University, Finland). The Finnish Twin Cohort dataset used in the current study will be located in the Biobank of the Finnish Institute for Health and Welfare, Finland. All the biobanked data are publicly available for use by qualified researchers following a standardized application procedure (https://thl.fi/en/web/thl-biobank/for-researchers accessed on 28 September 2022). The R packages used have been described in the *Methods* section. The R scripts have been deposited on github (https://github.com/gdrouard/Eval_MLclassifiers_CVDoutcomes).

## Abbreviations

CVD: Cardiovascular disease
ML: Machine learning
AE: Autoencoder
SBP: Systolic blood pressure
DBP: Diastolic blood pressure
LVDD: Left ventricular diastolic dysfunction
YFS: Young Finns Study
NMR: Nuclear Magnetic Resonance
E/e' ratio: Mitral peak velocity of early filling to early diastolic mitral annular velocity
E/A ratio: Ratio of the early to late ventricular filling velocities
LAVI: Left Atrial Volume Index
FTC: Finnish twin cohort
BMI: Body mass index
SSAE: Semi-Supervised Autoencoder
1LP: 1-Layer perceptron
LeakyReLU: Leaky Rectified Linear Unit
MSE: Mean Square Error
USAE: Unsupervised Autoencoder
CW: Connection Weights
1-sd classes: Algorithm; 1-standard deviation classes
sd: Standard deviation
svm: Support vector machine
GBM: Gradient boosting machine
VCDNs: View Correlation Discovery Networks
CoDT: Cross-omics Discovery Tensor
BCAAs: Branched-chain amino acids
MLP: Multi-layer perceptron
AUC: Area Under the Curve
rf: Random forest
HDL: High-density lipoprotein
VLDL: Very-low-density lipoprotein
