# Supplementary material for: Exploring machine learning strategies for predicting cardiovascular disease risk factors from multi-omic data
Source: BMC Med Inform Decis Mak. 2024 May 2;24:116. doi: 10.1186/s12911-024-02521-3 (PMC11064347; doi:10.1186/s12911-024-02521-3)
Supplement: Supplementary file 1 — Supplementary Material 1. [file 12911_2024_2521_MOESM1_ESM.pdf]

Supplementary document

Drouard et al.

last update: January 2023

ML-specific F1 performance for the target variables DBP, EERATIO, EARATIO and LAVI

DBP

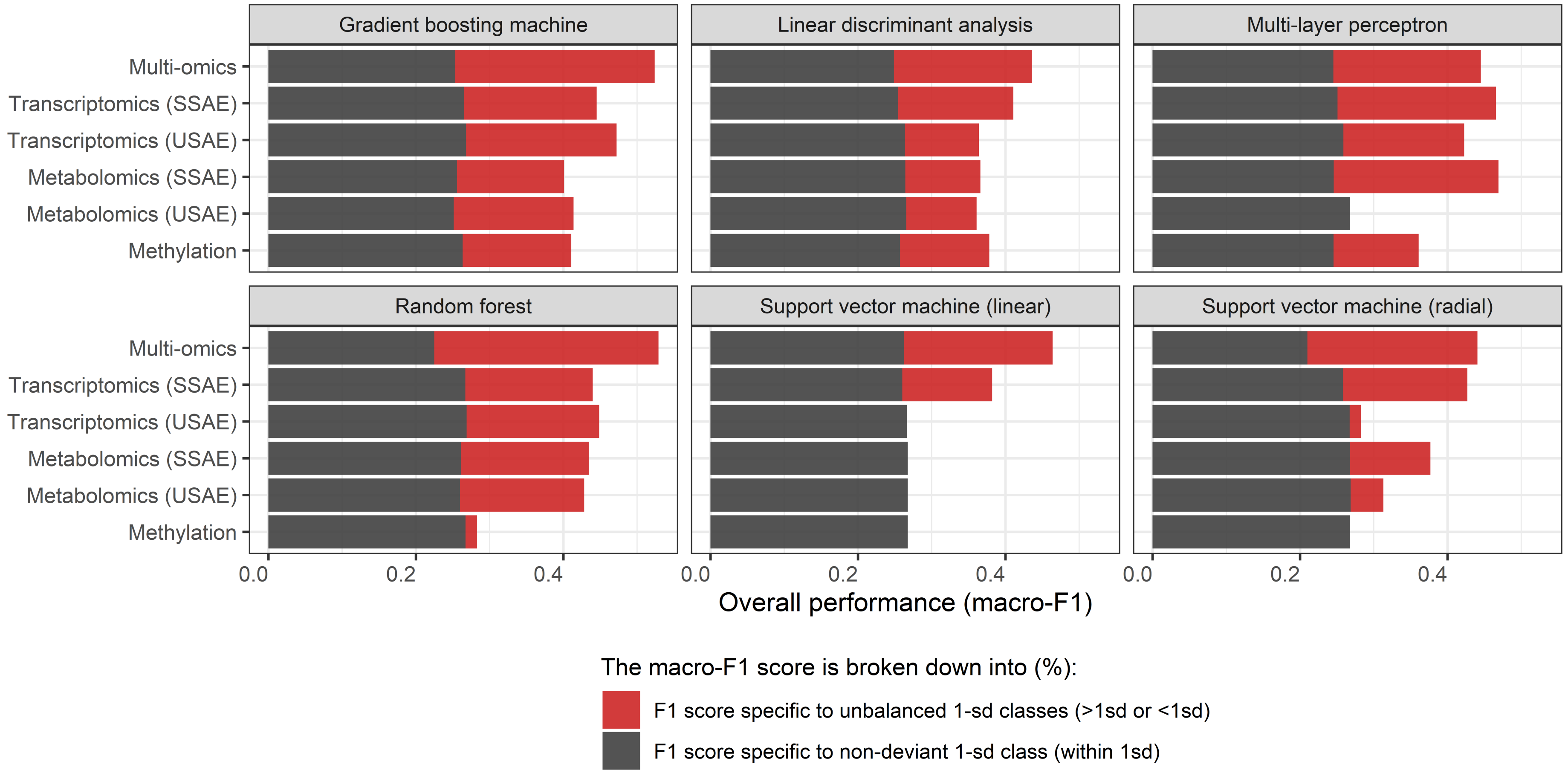

Figure S1: multi-method (cross-)omics performance for DBP prediction.

E/e' ratio

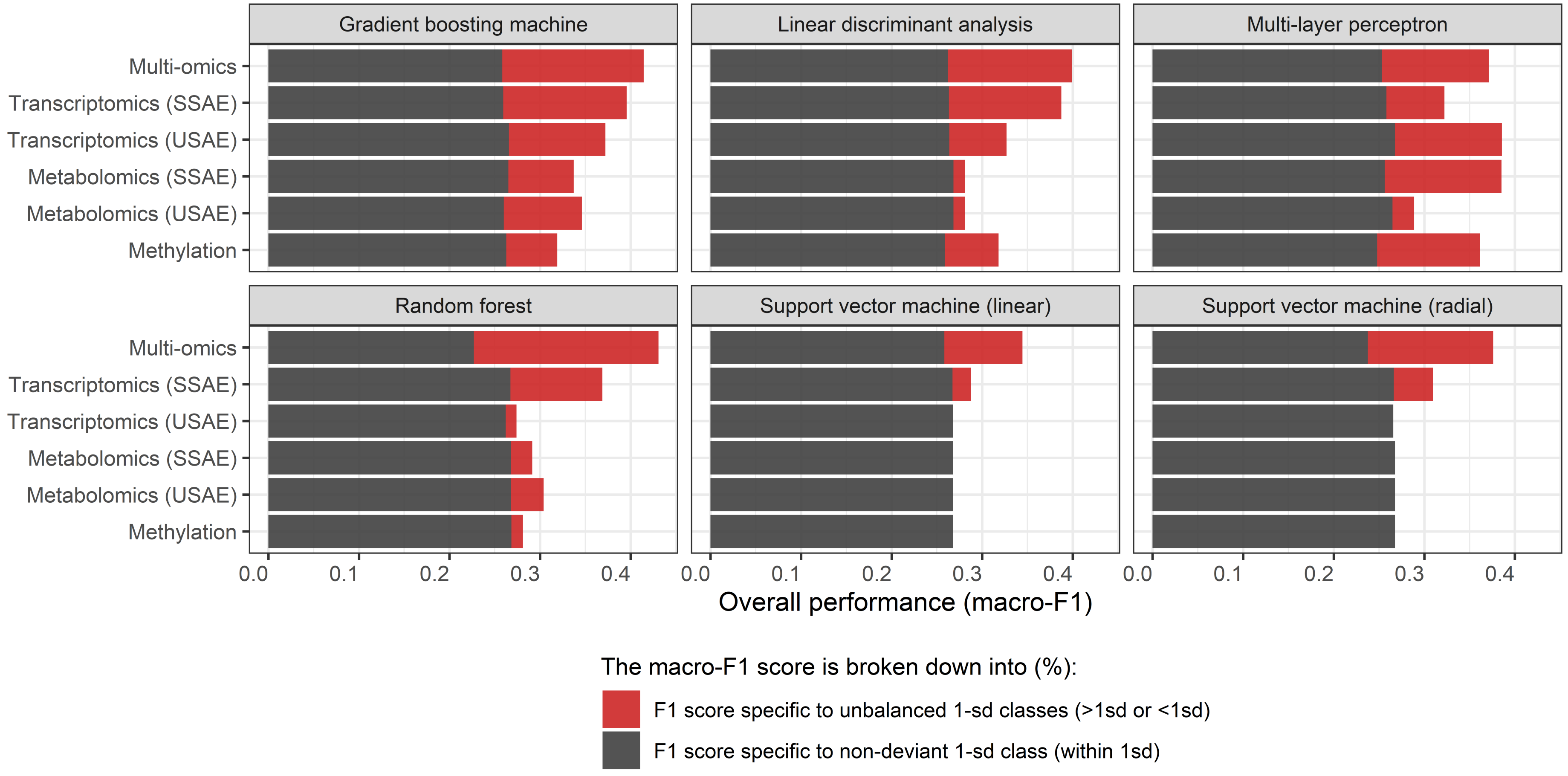

Figure S2: multi-method (cross-)omics performance for E/e' ratio prediction.

E/A ratio

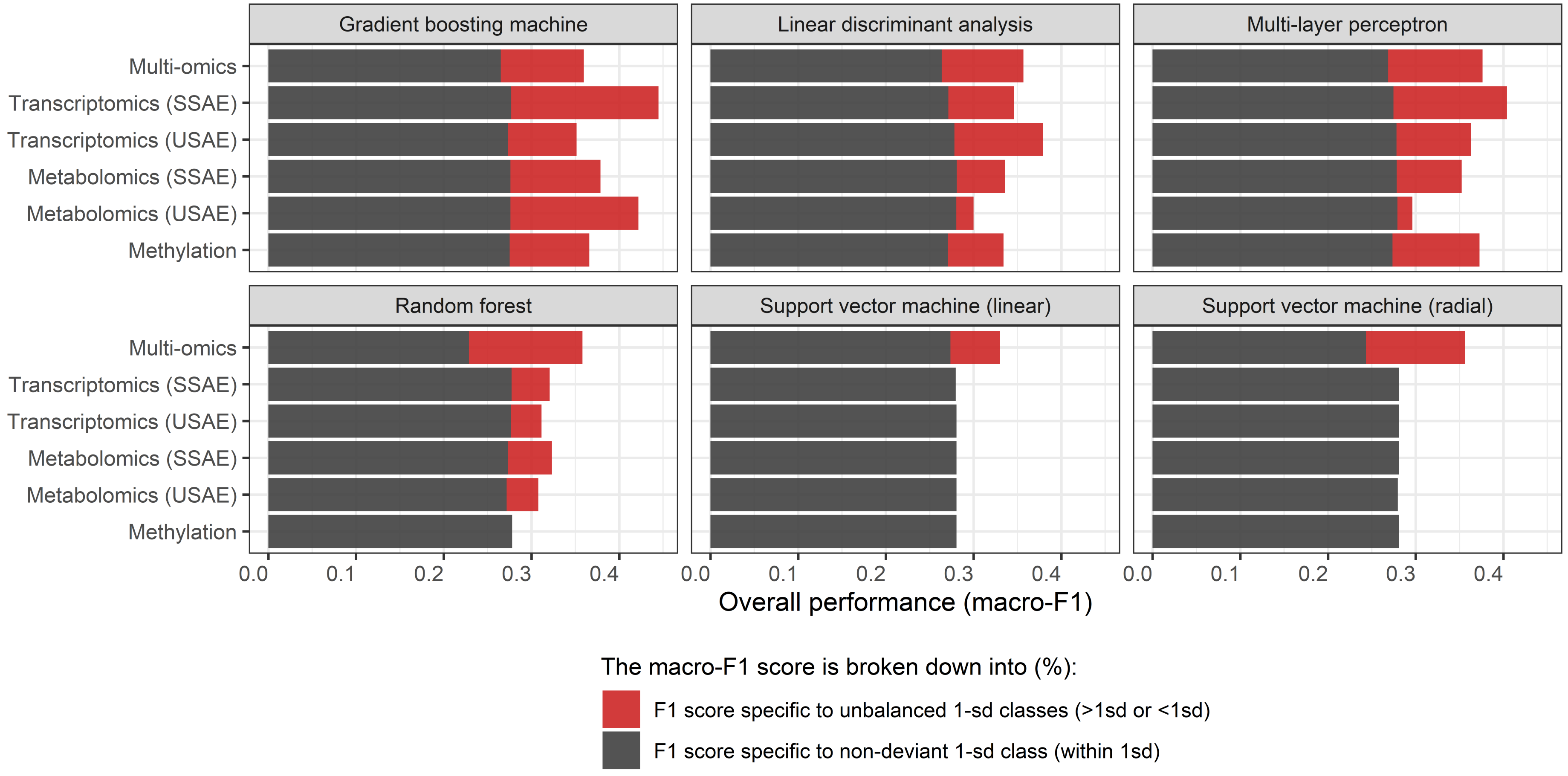

Figure S3: multi-method (cross-)omics performance for E/A ratio prediction.

LAVI

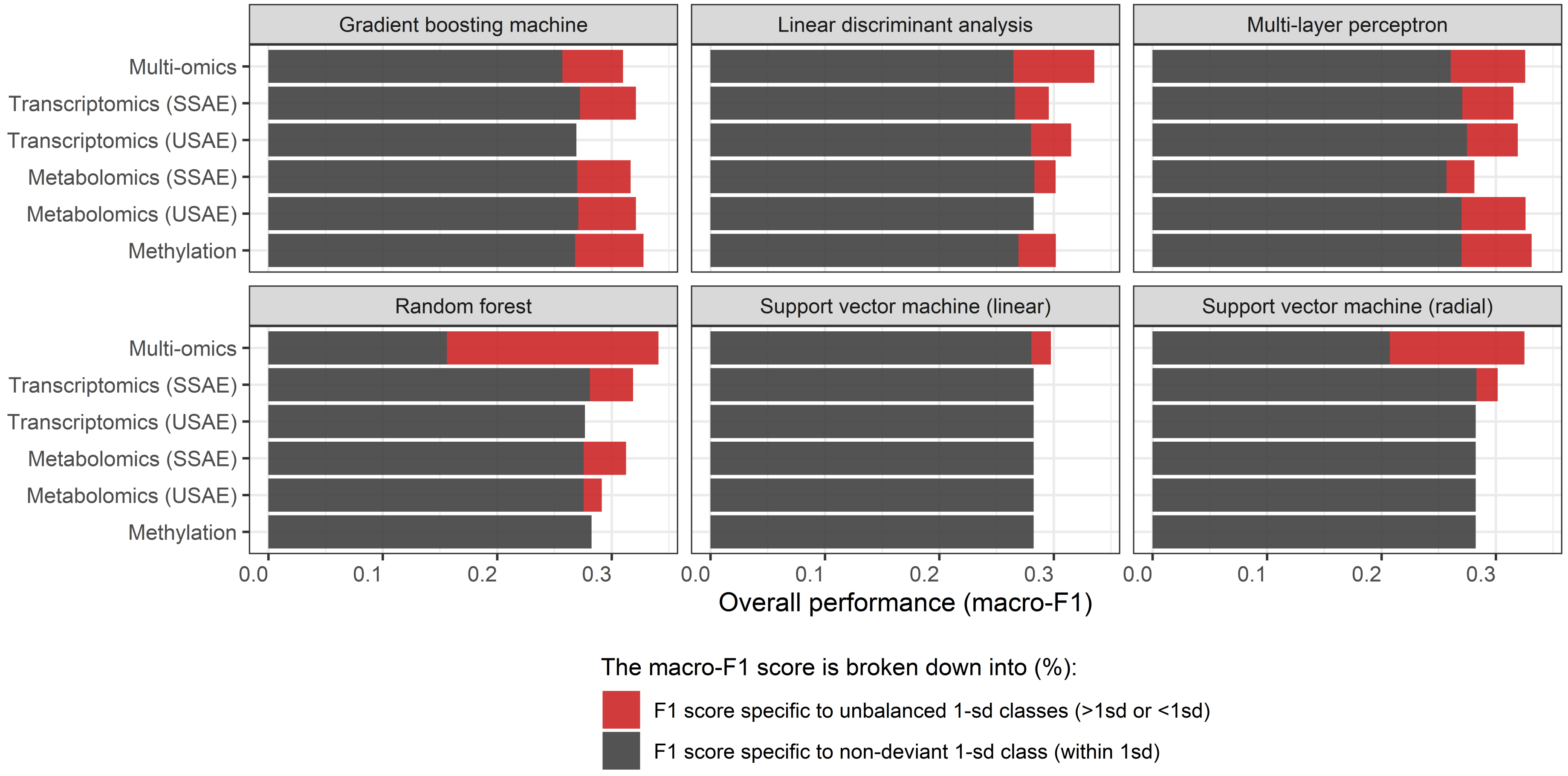

Figure S4: multi-method (cross-)omics performance for LAVI prediction.
